# Utilizing *Cymbopogon Proximus Grass* Extract for Green Synthesis of Zinc Oxide Nanorod Needles in Dye Degradation Studies

**DOI:** 10.3390/molecules29020355

**Published:** 2024-01-10

**Authors:** Manal A. Awad, Awatif A. Hendi, Khalid M. O. Ortashi, Reema A. Alnamlah, Asma Alangery, Eman Ali Alshaya, Saad G. Alshammari

**Affiliations:** 1 King Abdullah Institute for Nanotechnology, King Saud University, P.O. Box 2455, Riyadh 11451, Saudi Arabia; ealshaya@ksu.edu.sa; 2 Department of Physics and Astronomy, College of Sciences, King Saud University, P.O. Box 22452, Riyadh 11459, Saudi Arabia; awatifhendi@ksu.edu.sa (A.A.H.); 444204403@student.ksu.edu.sa (R.A.A.); 3 Department of Chemical Engineering, College of Engineering, King Saud University, P.O. Box 800, Riyadh 11421, Saudi Arabia; ortashi9@ksu.edu.sa; 4 Department of Chemistry, College of Sciences, King Saud University, P.O. Box 2455, Riyadh 11451, Saudi Arabia; aalangery@ksu.edu.sa (A.A.); salshammari@ksu.edu.sa (S.G.A.)

**Keywords:** green chemistry synthesis, *Cymbopogon Proximus*, zinc oxide nanorods-needles, physicochemical characteristics, photocatalysis, Rhodamine B

## Abstract

This study successfully synthesized zinc oxide nanorod needles (ZnO-NRNs) using an environmentally friendly method employing *Cymbopogon Proximus* extract. The resulting ZnO-NRNs exhibited exceptional physicochemical and structural properties, confirmed through various characterization techniques, including UV-Vis spectrophotometry, dynamic light scattering (DLS), transmission electron microscopy (TEM), X-ray diffraction (XRD), and energy-dispersive X-ray spectroscopy (EDX). The analysis revealed a hexagonal wurtzite structure with high crystallinity, a 3.6 eV band gap, and a notably blue-shifted absorption band. ZnO-NRNs showed impressive photocatalytic activity, degrading Rhodamine B dye by 97% under UV and visible sunlight, highlighting their photostability and reusability. This green synthesis process offers cost effectiveness and environmental sustainability for practical applications.

## 1. Introduction

The escalating severity of environmental pollution has spurred significant interest in innovative and high-performance photo-catalysts for pollutant degradation. However, the limited responsiveness of many catalysts to visible light remains a substantial challenge in achieving efficient photocatalytic conversion under natural sunlight irradiation [1].

To address these challenges and mitigate the detrimental impact of releasing unreacted chemicals into water bodies on the biosphere, various approaches are being explored, including the use of metal nanoparticles and microbial desalination cells. Semiconductor nanoparticles, particularly those with wide and direct band gaps, have gained significant attention as they play a vital role in the elimination of organic contaminants in wastewater [1].

Among these semiconductors, zinc oxide nanoparticles (ZnO NPs) have garnered substantial interest due to their versatility across a wide range of applications, spanning electronics, communications, sensors, and cosmetics, the biological and medical sectors, and environmental pollution treatment through adsorption or photo-degradation [2]. ZnO, characterized by its wurtzite-type crystal structure, high exciton binding energy (60 meV) at room temperature, and considerable direct bandgap (3.37 eV), offers unique properties for various applications [3].

The high binding energy of ZnO excitons allows for their existence at room temperature, resulting in a low laser emission threshold and significant natural emission radiative recombination yield. Furthermore, ZnO’s lack of a center of symmetry and exceptional electromechanical coupling contributes to its strong piezoelectric and pyroelectric capabilities, making it a preferred material for mechanical actuators and piezoelectric sensors [4,5]. Despite extensive research, a consensus on the emission mechanism of ZnO crystals still needs to be reached [6]. The elimination of antibiotics using photo-catalysis in sunshine and under ambient circumstances has attracted a lot of attention as a cost-effective, efficient, and ecologically friendly approach for removing antibiotic residues. Metal oxide-based photo-catalysts have lately garnered a lot of interest due to their excellent light absorption in the ultraviolet (UV), visible spectrum (VIS), or both, as well as their biocompatibility, safety, and stability across a wide range of conditions [7].

ZnO-NPs are valued for their proven photocatalytic, anticancer, antioxidant, and antibacterial properties [8,9]. In the field of photocatalysis, zinc oxide is a well-known n-type semiconductor. It has remarkable mechanical, electrical, and optical characteristics because of its high absorption efficiency and huge exciton binding energy. Biological and catalytic processes are only two of the many uses for ZnO-based materials. Pure ZnO nanoparticles are much sought after because of their remarkable photocatalytic activity [9]. In addition, it was found that ZnO nanoparticles may be made at a much cheaper cost than competing materials. To boost performance, however, dopants may be added to ZnO nanoparticles, and composite materials can be produced [10]. Traditional high-tech methods for nanoparticle production, such as chemical reduction, lithography, and beam epitaxy, often employ inexpensive but environmentally harmful materials [11,12]. In response, researchers have increasingly turned to green synthesis methods, harnessing natural resources and environmentally friendly processes to produce nanoparticles. Green chemistry, an emerging field, strives to minimize the use and release of hazardous chemicals, offering a more environmentally sustainable and cost-effective approach.

Using natural resources such as fruit, plant leaves, vegetables, and roots as raw materials, green synthesis has gained prominence in the creation of nanoparticles [13]. Among these resources, the genus *Cymbopogon* stands out for its numerous species with high essential oil content, found in the tropics and subtropics of Asia, Africa, and the Americas [14]. Historical evidence suggests the use of *Cymbopogon species* in traditional medicine, and various species have demonstrated pharmacological activities ranging from anticancer and cardioprotective to anti-inflammatory, antioxidant, antidiabetic, anticholinesterase, antibacterial, and antifungal properties [15,16,17].

*Cymbopogon Proximus*, known as Halfabar, Maharaib, and Alazkher, is of particular interest due to its strong fragrance and widespread distribution in Egypt and northern Sudan. Locals have long relied on this plant for its ability to induce smooth muscle relaxation, making it a popular choice for diuretic and antispasmodic purposes. Additionally, it has exhibited hypoglycemic, antipyretic, bronchodilatory, antibacterial, anticonvulsant, and antiemetic effects, further highlighting its pharmacological potential [18,19,20].

This research introduces a practical and sustainable approach for the eco-synthesis of zinc oxide nanorod needles (ZnO-NRNs) derived from a solution of zinc nitrate hexahydrate (Zn(NO_3_)_2_·6H_2_O). Utilizing an extract from *Cymbopogon Proximus* wild grass, we successfully synthesized ZnO-NRNs and subsequently investigated their optical properties and catalytic activity in the degradation of Rhodamine B (RhB) dye [21]. Conventional treatment methods often struggle with the breakdown of RhB, a challenging color contaminant frequently found in textile industry wastewater. Hence, RhB was selected as a representative pollutant to assess the photocatalytic efficiency. The primary goal of this study was to employ the synthesized ZnO-NRNs for dye degradation purposes. The synthesis and comprehensive characterization of ZnO-NRNs were carried out using appropriate methodologies. Subsequently, an evaluation was conducted to assess the biodegradability and detoxification potential of ZnO-NRNs in the degradation of RhB dye.

## 2. Results and Discussion

**Figure 1:**
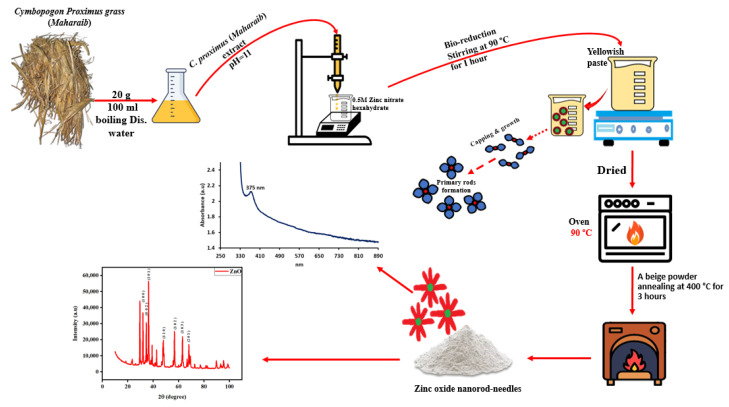
Schematic illustration of green synthesized ZnO-NRNs using an aqueous extract derived from *C. Proximus grass*.

**Figure 2:**
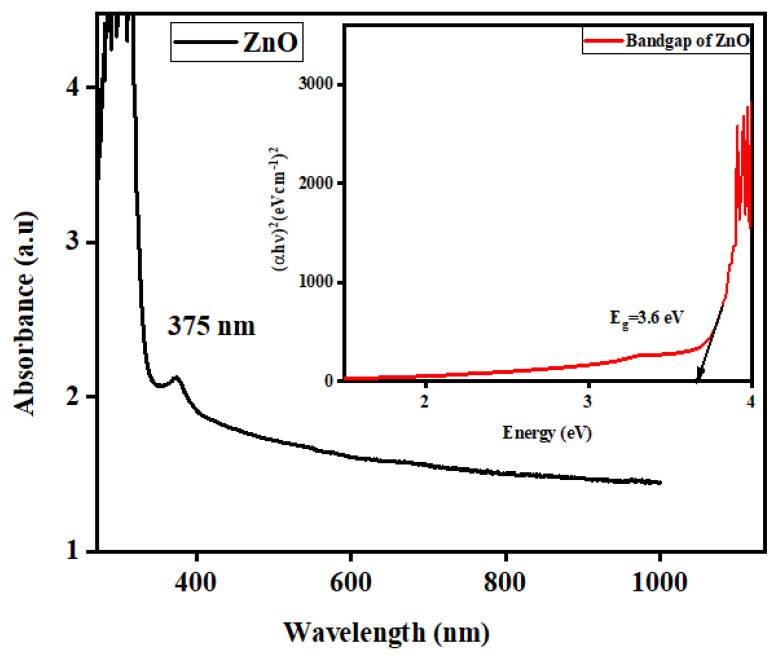
Absorption spectrum in the UV-visible range. The inset image is the optical band gap of the synthesized ZnO-NRNs.

**Figure 3:**
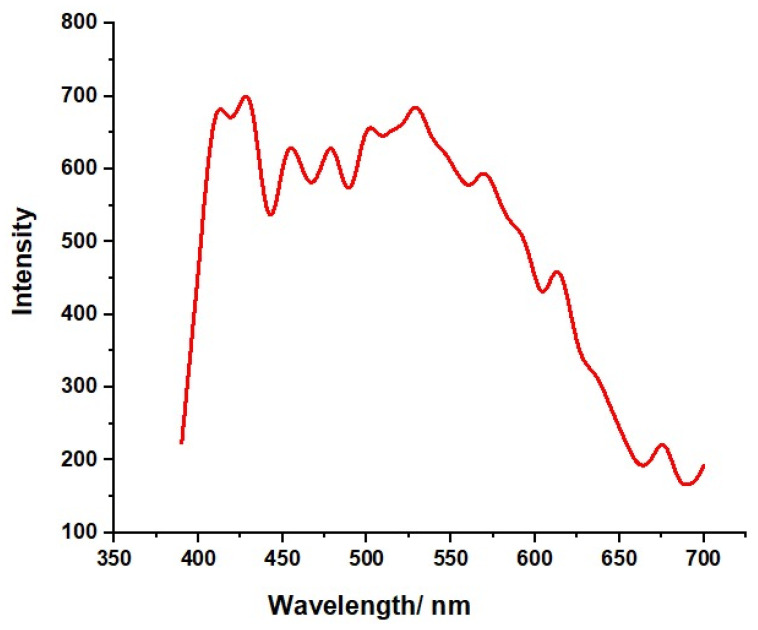
Photoluminescence spectrum analysis of synthesized ZnO-NRNs.

The production of nanoparticles in the *C. Proximus* extract was visually confirmed through the observation of lees and the presence of sediment in the reaction (Figure 1). The culmination of the reaction was marked by the formation of a white precipitate, as depicted in Figure 1. To delve into the optical characteristics of the synthesized zinc oxide nanorod needles (ZnO-NRNs), both UV absorbance and photoluminescence (PL) spectra were meticulously examined, as shown in Figure 2 and Figure 3, respectively. The existence of secondary metabolites in plants leads to the transformation of the zinc ions in the solution into zinc oxide. The plant extract serves not only as a reducing agent but also as a stabilizing agent. The confirmation was obtained using UV-visible spectrum analysis within the wavelength range of 280 nm to 800 nm. UV-vis spectroscopy is a well-established technique for the analysis of size- and shape-controlled nanoparticles in aqueous solutions, allowing for the measurement of the wavelengths of light absorbed by the nanoparticles. In our investigation, the prepared ZnO-NRNs were subjected to absorbance spectra analysis spanning a range from 250 to 1000 nm. The distinctive high UV absorption spectrum displayed by the synthesized sample can be attributed to surface plasmon resonance, with a notable absorption peak observed at 375 nm (Figure 2) [22]. To determine the optical band gap of the ZnO-NRNs, we employed the Tauc plot technique, utilizing UV-visible spectra data. This technique involves extrapolating a straight line between the absorption coefficient (αhν)^2^ and the corresponding energy levels (eV). Our analysis yielded an optical band gap value of 3.6 eV, which is in close agreement with the existing literature [1,7].

Furthermore, our findings underscore the high degree of phase purity in the ZnO NRs, which is consistent with prior research [2]. Notably, the absorption peaks underwent redshifts to longer wavelengths due to electron excitation from the valence band to the conduction band [22]. Gunalan Sangeetha et al. elucidated that as particle size increases, metal oxide nanoparticles exhibit changes in their optical absorption spectra towards longer wavelengths, reflecting their unique optical properties associated with surface plasmon resonance (SPR). The precise wavelength and location of plasmon band absorption are influenced by factors such as nanoparticle size and shape, the dielectric constant of the surrounding medium, and the species adsorbed on the nanoparticle surface. It is also worth noting that unlike anisotropic nanoparticles, spherical nanoparticles, in accordance with Mie’s theory, typically exhibit a single SPR band. As the symmetry of a nanoparticle deviates from sphericity, additional SPR peaks become apparent [23,24,25,26].

Photoluminescence (PL) investigations were conducted to highlight the emission characteristics, as seen in Figure 3. Photoluminescence spectroscopy plays a vital role in the investigation of surface defects, contaminants, and energy bands of materials [27]. In the case of ZnO, near band-edge (NBE) emission is primarily attributed to the radiative recombination of free excitons [28,29]. To delve into the photoluminescence characteristics of the synthesized ZnO-NRNs, we conducted spectroscopy at room temperature across a wavelength range of 300–700 nm, with an excitation wavelength of 375 nm [30]. The resulting photoluminescence spectrum (Figure 3) exhibits numerous emission peaks within the central wavelength range of 410–680 nm. The presence of plant extracts appears to influence certain physical properties of ZnO crystal surfaces. In the absence of significant peaks in the green emission region, pure ZnO crystals display strong emission at 412 nm due to intrinsic factors. ZnO crystal defect structures correspond to the blue band at 412 nm. The recombination of oxygen sources within the ZnO crystal likely accounts for this phenomenon [31,32]. In the case of the nanoparticles, the primary green emission peak was observed at 568.9 nm. This peak corresponds to the recombination of electrons from oxygen vacancies with holes from zinc vacancies and exhibits a broad and robust profile. It is worth noting that the energy level splitting of plant extracts in the ZnO host lattice is significantly influenced by the surrounding ligand field [33]. This maximum value may be associated with the band gap of ZnO-NRNs and the absorption peaks observed in UV-Vis measurements. The 2.38 eV (around 522 nm) green band is attributed to the radiative recombination of photogenerated holes and electrons from singly ionized vacancies in the surface and subsurface [34,35]. Consequently, it was suggested that multiple centers may concurrently contribute to green luminescence. This is due to the inherent imperfections and point defects in zinc oxide crystals, which can take on various forms [36,37].

**Figure 4:**
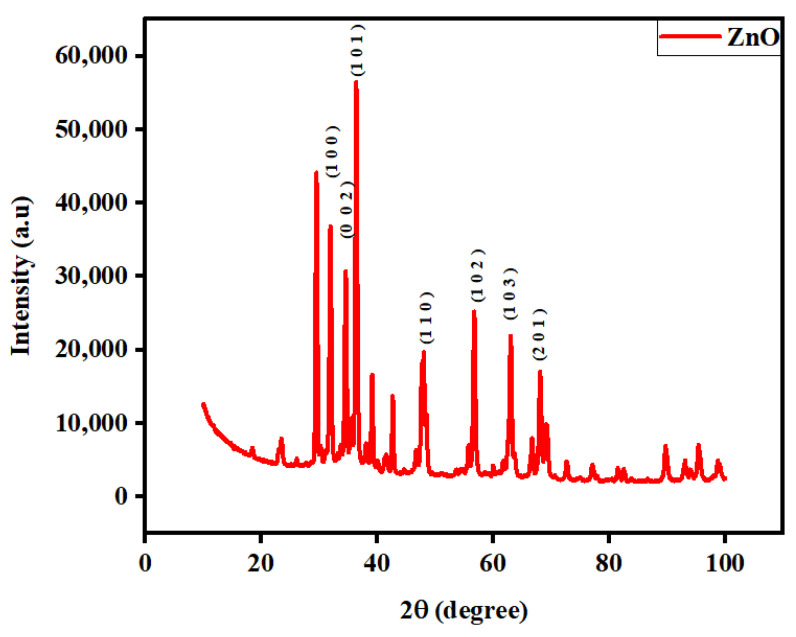
2Theta of XRD spectrum of green-synthesized ZnO-NRNs from *Alazkher* or *Maharaib*.

The X-ray diffraction (XRD) pattern, as illustrated in Figure 4, provides valuable insights into the structural characteristics of the synthesized ZnO-NRNs. The pattern clearly reveals the characteristic hexagonal wurtzite structure, which is typical for pure ZnO nanorods. The most prominent diffraction peaks were observed at 2θ values of 31.819°, 34.523°, 35.625°, 47.549°, 56.675°, 62.837°, 67.855°, 69.181°, 89.643°, and 95.415°. These values correspond to specific lattice planes, denoted by their Miller indices, including (100), (002), (101), (110), (102), (103), (201), (203), and (311) (COD 2300113). The sharp and well-defined nature of these diffraction peaks is indicative of the high crystalline quality of the material [38]. Notably, there are very small discernible peaks (at 39° and 42°) associated with possible dust contaminants [39,40], indicating synthesis of pure ZnO-NRNs. The inclusion of secondary metabolites appears to have a positive impact on the crystalline development of ZnO-NRNs, further confirming the absence of impurities. The comprehensive diffraction analysis performed aligns excellently with the JCPDS#36-1451 reference for the hexagonal wurtzite crystal structure of ZnO-NRNs, providing robust evidence for the successful formation of this crystal structure. These findings are consistent with previous research in the literature [41,42,43,44].

Additionally, various analyses—including the determination of crystal lattice indices, evaluation of crystallinity, and calculations of average crystallite size and interplanar d-spacing—were conducted further to characterize the structural properties of the synthesized ZnO-NRNs. All the peaks in the diffraction curves that stand in for the reflection planes allowed us to determine the lattice constant, where a = b = 3.452 (Å) and c = 55.3 (Å).

Using the Debye–Scherer Equation (1),

(1) D (nm)=kλ/βCosθ

where *D* represents the size of the crystallite, *k* is the form factor with a value of 0.9, *λ* is the wavelength of 0.15418 nm (CuK), *β* represents the full width at half maximum (FWHM), and *θ* signifies the diffraction angle. The typical crystal size was determined to be approximately 35 nm, and the following formula was used to calculate the interplanar spacing:

(2) $$d=\frac{n\lambda }{2}Sin\theta$$

where *d* represents the interplanar spacing, *n* represents the order, *λ* is the wavelength of 0.15418 nm (CuK), *θ* signifies the diffraction angle, and the typical spacing was determined to be approximately 2 nm.

**Figure 5:**
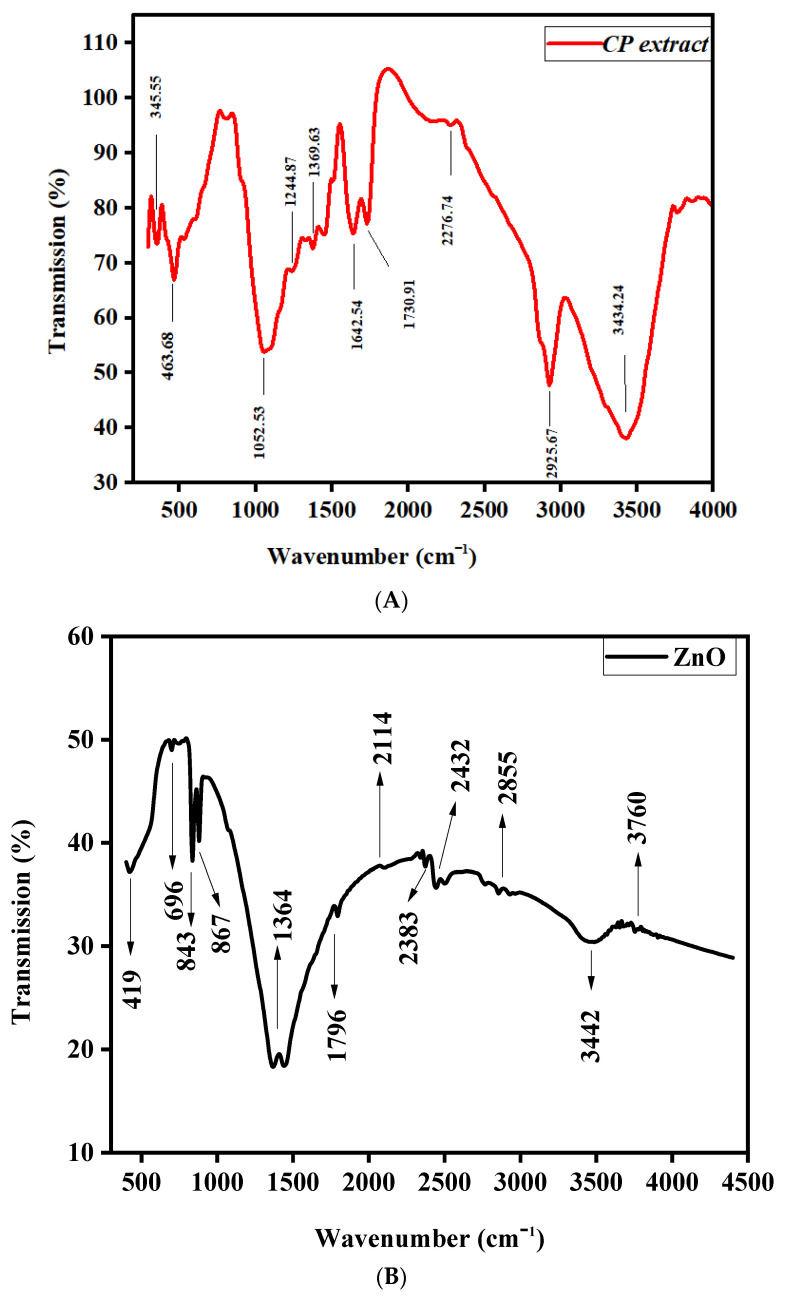
FTIR spectrum of (**A**) *C. Proximus grass* extract and (**B**) green-synthesized ZnO-NRNs using an aqueous extract derived from *C. Proximus grass*.

Functional groups in *C. Proximus grass* extract and ZnO-NRNs were examined using FTIR within the wavelength range of 500–4500 cm^−1^ (refer to Figure 5 (Figure 5A and Figure 5B, respectively). The functional group formation and chemical composition of synthesized ZnO nanoparticles can be determined with FTIR. It also indicates that the interaction between the phenolic chemicals, alkynes, terpenoids, and flavonoids is responsible for the existence of ZnO nanoparticles. In order to determine which molecules may be responsible for capping and efficiently stabilizing the metal nanoparticles synthesized by green approach, Fourier transform infrared spectroscopy (FTIR) spectra of aqueous zinc oxide nanoparticles is carried out from aqueous extract taken from *C. Proximus grass*. The bands were formed when zinc ions were reduced to ZnO by the functional groups. Different stretching modes are represented by different bands. According to Figure 5A,B, the hydroxyl alcohol group’s (O-H) fundamental mode of vibration is at 3442 and 3434.24 cm^−1^, symmetric alkenes (C≡C) are at 2114 and 2276.74 cm^−1^, (C-C) aromatic stretching is at 1796 and 1730.91 cm^−1^, and (C-N) amine C-N stretching is at 1364 and 1369.63 cm^−1^, for ZnO-NRNs and the extract, respectively [45]. The presence of the alkene group was identified at the wavenumber of 1796 cm^−1^. Additionally, the stretching vibrations of alcohols/carboxylic acids were observed at the wavenumber of 867 cm^−1^ (C–O), as depicted in Figure 5B. Furthermore, the characteristic band corresponding to the Zn–O bond, which serves as confirmation of the material being zinc oxide, was observed in the wavenumber range of 696–419 cm^−1^, indicating the Zn–O stretching vibration [46,47].

**Figure 6:**
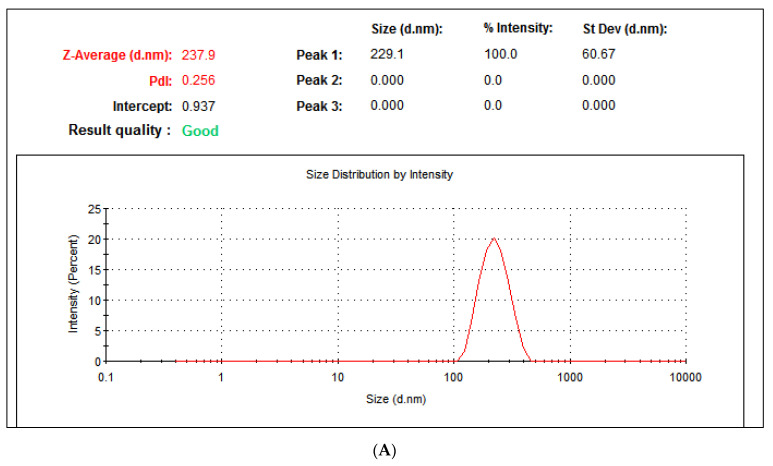

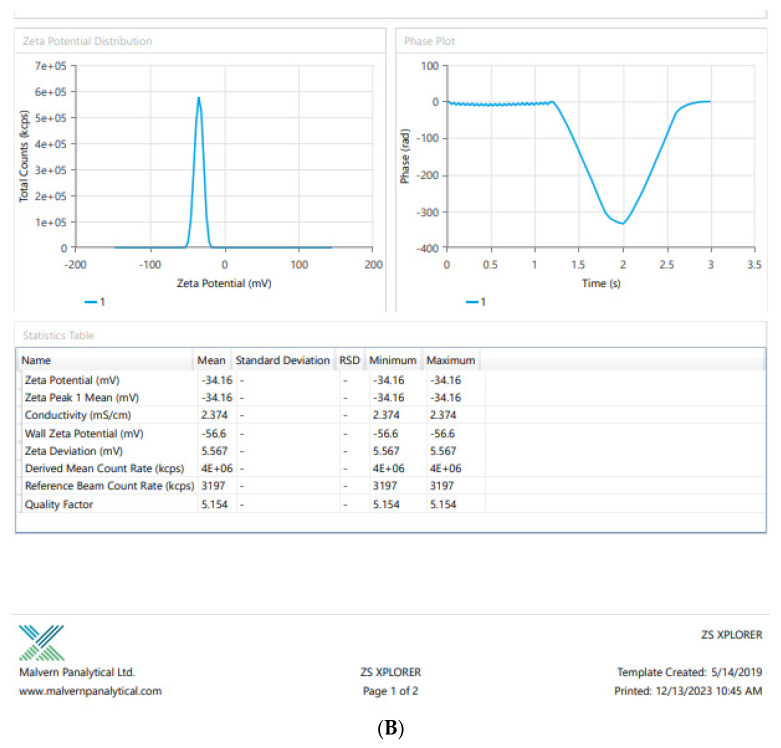
(**A**) DLS and (**B**) Zeta potential analysis of ZnO-NRNs from *Alazkher* or *Maharaib*.

Dynamic light scattering (DLS) is a widely employed technique that relies on the Brownian motion of particles in a suspension to determine the hydrodynamic diameter of nanoparticles. In our investigation, DLS analysis was conducted to estimate the average hydrodynamic size of the nanoparticles, as illustrated in Figure 6A. It is worth noting that the DLS-calculated average hydrodynamic size of the nanoparticles appears to be significantly larger than the theoretical size calculated via X-ray diffraction (XRD) [48]. This disparity in size estimation may be attributed to the fact that nanoparticles often exist in the form of aggregates or agglomerates, which can lead to variations in observed size. In our study, the DLS analysis revealed that the produced ZnO-NRNs exhibited an average particle size distribution of 237.9 nm when measured by intensity. The intercept of this distribution was calculated to be 0.937. Importantly, the nanoparticles in the medium exhibited a Polydispersity Index (PDI) of 0.256. This PDI value is indicative of the monodispersity and homogeneity of the nanoparticles, suggesting a relatively uniform size distribution [49]. Zeta potential evaluation serves as a key indicator of the colloidal stability of nanoparticles in a solution, hinging on their surface charge. Negative or positive Zeta potential values demonstrate the nanoparticles’ ability to repel each other, thereby preventing aggregation [48]. In this study, the measured Zeta potential value for synthesized ZnO-NRNS was determined to be −34.16 mV, suggesting a high level of stability in water (refer to Figure 6B). This outcome aligns with findings from previously reported studies as [50,51,52,53].

**Figure 7:**
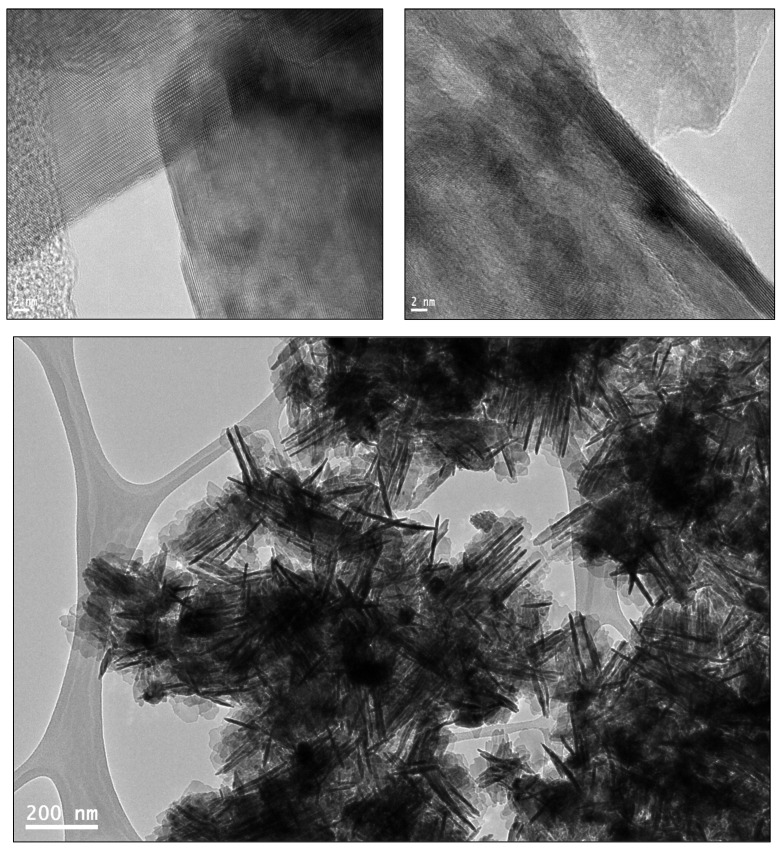
TEM macrophages of ZnO-NRNs from *Alazkher* or *Maharaib*.

**Figure 8:**
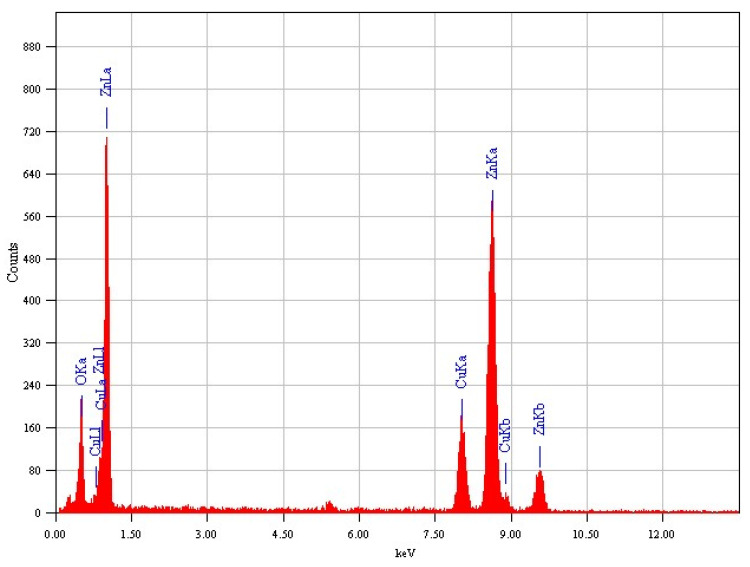
EDX spectrum analysis of the ZnO-NRNs from *Alazkher or Maharaib*.

The investigation of surface morphology and elemental composition of the synthesized nanorods using plant extract was carried out through transmission electron microscopy (TEM) and energy-dispersive X-ray (EDX) spectroscopy, shedding light on the structural and compositional aspects of these materials. As depicted in Figure 7 and Figure 8, respectively, the ZnO-NRNs (produced via a green synthesis approach) are clearly visible in the form of rods and needles. Notably, the crystallite size, as determined with XRD analysis, was found to be smaller than the particle size revealed through morphological examination. This suggests that the unique combination of biomolecules present in the *Alazkher grass* extract plays a pivotal role in achieving the distinctive nanoparticle shape observed in this study, setting it apart from other systems [54].

Traditionally, the production of ZnO nanorods often involves the use of strong bases, chemical surfactants, or capping agents. However, in this study, the *Alazkher* extract was employed as a substitute for these compounds, offering a greener alternative. Importantly, the extraction of phytochemicals from the plant does not necessitate the use of any organic solvents. It is noteworthy that a comparable nanostructure can be achieved using a green extract, making the synthesis process more cost-effective and environmentally friendly. This underscores the potential of plant-based extracts as a sustainable and eco-friendly route for nanomaterial synthesis.

To ascertain the elemental composition of the synthesized zinc oxide nanoparticles, an energy-dispersive X-ray (EDX) analysis was meticulously conducted. Figure 8 presents the EDX spectrum obtained in spot-profile mode, focusing on a region rich in nanoparticles during their fabrication. The spectrum distinctly reveals the presence of only zinc and oxygen elements, as evidenced by the presence of two distinct peaks—one corresponding to zinc and the other to oxygen. This observation unequivocally confirms the exceptional purity of the ZnO-NRNs, with no indications of other contaminants. The elemental profile further corroborates the synthesis of ZnO-NRNs, as indicated by three distinct peaks between 1 and 10 keV, with a prominent peak at around 1 keV, which is characteristic of zinc. The faint Cu signal originates from the copper grid used in the analysis. Importantly, there are no impurity peaks, and the concentrations of oxygen and zinc are found to be stoichiometric. This consistency in the increase of Zn in the EDX spectrum aligns with findings reported by several sources in the literature [55,56,57,58]. It is worth noting that the surface plasmon resonance of zinc oxide nanoparticles is responsible for their optical absorption peaks, as demonstrated with the EDX analysis [59]. Nanoparticles devoid of impurities hold significant promise across a wide range of application sectors, including photo-catalysis, due to their exceptional purity and composition.

The assessment of ZnO-NRNs’ efficacy as a photocatalyst for degrading Rhodamine B (RhB) dye, both under visible and UV light, was carried out using Equation (3).

DE% = (A_0_ − A)/A_0_ × 100 (3)

where A_0_ is the initial absorption intensity, and A is the absorption intensity after photo-degradation occurs entirely.

**Figure 9:**
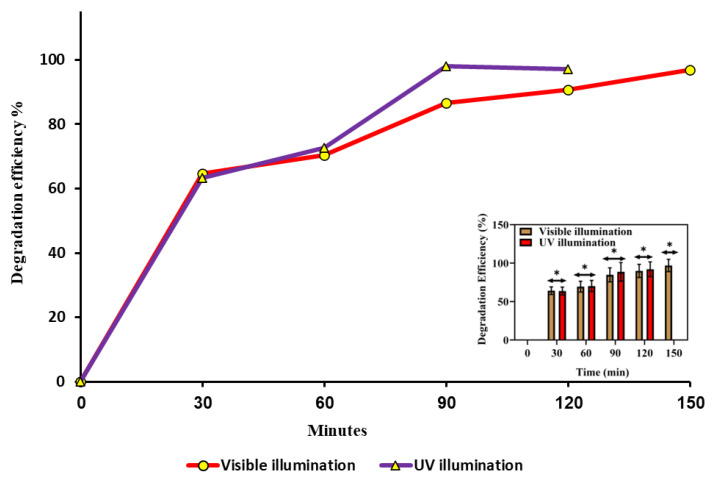
ZnO-NRNs degrade RhB dye under visible and UV light, increasing efficiency with time (The data are presented as mean ± SD of three experiments. The data were analyzed statistically by one way analysis of variance (ANOVA) followed by Dunnett’s Multiple range test (Tukey’s post-hoc test) using GraphPad Prism software. Significance “*” represent *p* < 0.05 respectively).

The degradation of dye solutions over various periods is illustrated in Figure 9 for visible and UV light irradiation. As the irradiation period lengthened, the peak intensity of the dye gradually diminished, signifying the degradation process. Notably, the finding results reveals that the degradation efficiency of the dye solutions was more pronounced under UV light compared to visible light. When ZnO-NRNs were employed as a catalyst for RhB (10 mg/L) degradation in water, exposure to natural sunlight for 16 h or ultraviolet light for 160 min resulted in nearly complete degradation (97%), as demonstrated in Figure 9.

The lower degradation efficiency of organic compounds under visible light, as compared to UV light, can be attributed to the relatively larger bandgap energy of ZnO-NRNs [60,61,62,63]. The larger bandgap energy necessitates higher-energy photons, which are predominantly present in the UV spectrum, to initiate photocatalytic reactions. This finding underscores the importance of light source selection when utilizing ZnO-NRNs as photo-catalysts for organic compound degradation. Separation of photo-generated electron–hole pairs is further enhanced when ZnO is combined with additional materials to create a hybrid photo-catalyst system. First and foremost, nanostructured ZnO is classified in zero, one, two, and three dimensions, with further subdivision into planar, dots, and quantum arrays. The greater specific surface area of ZnO, as reported by [64], facilitates the adsorption of additional contaminants during the photocatalytic degradation process. ZnO’s poor crystallinity also aids in the separation process by trapping photo-induced electron–hole pairs.

Heterogeneous photocatalysis operates on the concept that natural or artificial light may activate a semiconductor. An electron–hole pair (eCB + hVB+) is created when solar radiation hits ZnO-NRNs. This process involves the transfer of electrons from the valence band to the conduction band, as follows:

ZnO + *hν* (solar radiation) ⟶ ZnO (e_CB_^−^ + h_VB_^+^) (4)

Symbols represent both the conduction electrons and the valence electron holes as eCB− and hVB^+^, respectively. The electrons created during photoexcitation have the potential to engage in reactions with acceptor electrons. These acceptor electrons may originate from oxygen molecules that are dissolved in the liquid medium around the semiconductor. As a result of this interaction, superoxide anions are produced (^•^O_2_^−^) as follows:

O_2_ + e_CB_^−^ ⟶ ^•^O_2_^−^ (5)

The oxidation of OH- by photogenerated holes yields ^•^OH:

OH^−^+ h_VB_^+^ ⟶ ^•^OH (6)

The roughness of the ZnO-NRN surface is responsible for the adsorption of RhB molecules and the subsequent sensitization of the ZnO photocatalyst. RhB molecules readily attain an excited state because they are sensitive chromophores that absorb light over a broad range of wavelengths, including solar energy. Excited RhB may readily inject its electrons into the ZnO CB, resulting in an RhB molecule with a positive charge (RhB^+^). As discussed below, RhB degrades in the presence of solar light by self-sensitized photolysis.

RhB + *hv* = RhB* (7)

RhB* + ZnO⟶ ZnO (e_cb_^−^ + h_VB_^+^) +RhB^+^ (8)

Degradation and mineralization products are formed when the active species (^•^OH, ^•^O_2_, and h^+^) oxidize RhB dye molecules:

^•^O_2_^−^ + RhB/RhB* ⟶ Degradation products⟶ CO_2_ + H_2_O (9)

^•^OH + RhB/RhB* ⟶ Degradation products⟶ CO_2_ + H_2_O (10)

h_VB_^+^ + RhB/RhB* ⟶ Degradation product⟶ CO_2_ + H_2_O (11)

**Figure 10:**
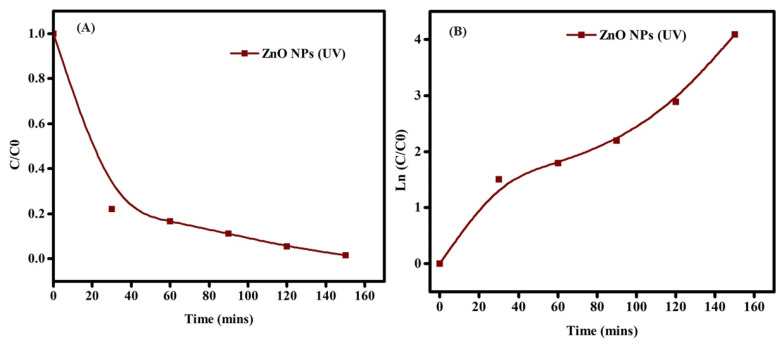

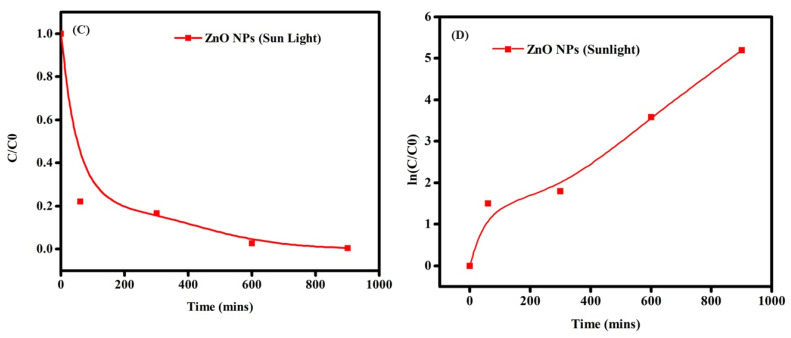
The photocatalytic performance of RhB was assessed through (**A**,**C**) analysis of the C/C_0_ plot and (**B**,**D**) examination of the apparent degradation rate constants under UV and visible light radiation, respectively.

Degradation may occur by photooxidation (generating hydroxyl radicals) and photoreduction (generating peroxide radicals). This mechanism aligns with previous research findings [65,66,67,68,69,70,71]. Using the first-order kinetic equation, we can evaluate the process rate (Figure 10).

ln(C_0_/C_t_) = kt (12)

**Figure 11:**
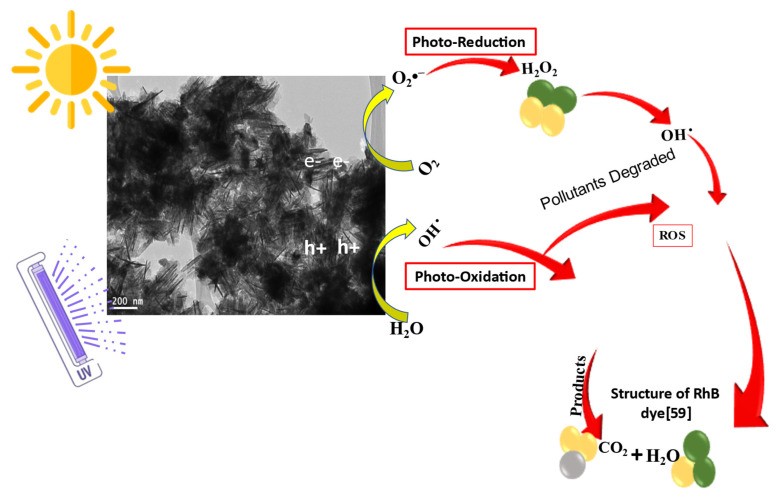
Possible mechanism of photocatalytic degradation of RhB dye with ZnO-NRNs.

The symbols C_0_ and C_t_ represent the starting and final concentrations of ZnO-NRNs, respectively, while t denotes the degradation period and k signifies the rate constant. The dye degradation rate constants were measured to be 0.021913 min^−1^ and 0.155 min^−1^ in the presence of UV and visible light, and half-life are depicted in Table 1**.** Ultimately, this reactive oxygen species (ROS) facilitated the degradation of the dye compound, forming mineral acids, carbon dioxide (CO_2_), and water. The likely mechanism is shown in the inset of Figure 11.

Ultimately, these reactive oxygen species (ROS) facilitate the degradation of the dye compound, leading to the formation of mineral acids, carbon dioxide (CO_2_), and water. The likely mechanism is depicted in the inset of Figure 11.

When contrasting our results with those of analogous studies involving ZnONPs-based photocatalysts synthesized through diverse methods and reported in the literatures, the synthesis of ZnONPs using *Cymbopogon Proximus* extract emerges as noteworthy. Notably, our findings reveal a superior degradation percentage compared to other reported instances, as indicated in Table 2.

## 3. Experimental Methods

### 3.1. Green Synthesis and Characterization of Zinc Oxide Nanorod Needles

Twenty grams of *C. Proximus* (*Alazkher or Maharaib grass*) was washed twice with regular tap water and then distilled. After the *Alazkher* or *Maharaib grass* was exposed to air for roughly a day beneath a hood, it was crushed into a fine powder. The liquid extract was prepared by dissolving 200 mg of the powder in 100 mL of hot, distilled water and letting the mixture sit at room temperature overnight. The isolated liquid underwent further filtering.

*The Alazkher* or *Maharaib* extract (pH = 11) was mixed with 0.5 M zinc nitrate hexahydrate (Zn(NO_3_)_2_·6H_2_O) (bought from Sigma-Aldrich, Stockholm, Sweden) and stirred at 90 °C for 1 h to create a brownish paste. The liquid was then removed from the sediment by centrifuging at 15,000 rpm for 5 min. The particles formed after precipitation underwent several washes in ethanol and then distilled water to flush out any remaining contaminants. The resultant paste, a beige powder consisting of ZnO-NRNs, was obtained after 5 h of drying at 70 °C and then annealed at 400 °C for 3 h [21].

Several different types of equipment were used to characterize the ZnO nanorods.

Using a Bruker D8 ADVANCE X-ray diffractometer (Bruker, Billerica, MA, USA) set to 40 KV and 40 MA with CuKa radiation at 1.5418, the crystalline structures of ZnO-NRs were analyzed. Using a transmission electron microscope (TEM; JEM-1400; JEOL; Tokyo, Japan), the size and form of the particles were examined. The light absorbance, optical properties, and bandgap energy of the synthesized ZnO-NRs were estimated using their UV-vis absorption spectra (Shimadzu-1800) from 200 to 900 nm. A JEM-2100F TEM was used for EDX analysis to identify the fundamental building blocks of the suspended ZnO-NRs. The hydrodynamic diameter and polydispersity index (PDI) of colloidal synthesized ZnO-NRNs were determined using dynamic light scattering (DLS) with a Zetasizer (HT Laser, ZEN3600, Malvern Nano Series, Instruments, Malvern, UK). Additionally, Zeta potential analysis was conducted using Zetasizer Advance (ZSU3305, Malvern Nano Series, Instruments, UK). The identification of functional groups on the surface of ZnO NPs and within the extract was carried out using a Fourier transform infrared (FTIR) spectrometer (Shimadzu IR, Prestige 21, Nakagyo-Ku, Japan). The scan covered a range of 4000–400 cm^−1^ and the analysis took place at the Chemistry Department of King Saud University, Female Students Campus, Riyadh, Saudi Arabia.

### 3.2. Photocatalytic Activity Study

Rhodamine B (RhB) was used to test the produced sample’s photocatalytic degradation capacity under UV light and solar irradiation [22]. A cuvette suitable for use in a laboratory was filled with 20 mL of dye solution, and the nanoparticle sample was distributed within. Afterward, we placed the concoction in front of a UV lamp or let it bake in the sun. Using a UV/Vis spectrophotometer, optical absorption spectra were calculated for various light exposure times. Degradation was observed by measuring the dye’s decreasing maximum wavelength absorption intensity.

The degradation efficiency (DE%) was calculated using the following equation:

DE% = (A_0_ − A)/A_0_ × 100 (13)

where A_0_ is the initial absorption intensity, and A is the absorption intensity after photo-degradation occurs entirely.

## 4. Conclusions

This study has successfully demonstrated the effective synthesis of zinc oxide nanoparticles (ZnO NRNs) using a straightforward and innovative green chemistry approach that employed *Alazkher* or *Maharaib* aqueous extract as a reducing agent. X-ray diffraction (XRD) analysis confirmed that the ZnO-NPs were synthesized in a single crystalline phase, exhibiting a hexagonal wurtzite structure with a high degree of crystallinity, as evidenced by sharp peaks. The average crystal size was determined to be approximately 35 nm, and the interplanar spacing was calculated to be around 2 nm. Transmission electron microscopy (TEM) images revealed the presence of nanoscale particles with rod and needle shapes in the ZnO samples. This morphological analysis further supported the successful synthesis of ZnO-NPs. The synthesized samples were found to be devoid of contaminants, with the presence of zinc and oxygen elements confirmed by both XRD and energy-dispersive X-ray (EDX) analyses. Dynamic light scattering (DLS) results indicated a Polydispersity Index (PDI) of 0.256 and Zeta potential of −34.16 mV for the ZnO-NRNs in the medium, highlighting their monodispersity, homogeneity, and high stability. The UV-visible absorbance of the synthesized ZnO-NPs was measured at 375 nm. Additionally, the photoluminescence spectrum displayed numerous emission peaks within the central wavelength range of 410–680 nm and a strong emission at 412 nm. The green band at 522 nm (2.38 eV) was linked to the radiative recombination of photogenerated holes and electrons from singly ionized vacancies in the surface and subsurface. Additionally, FTIR results revealed the characteristic band manifested in the wavenumber range of 696–419 cm^−1^, signifying the presence of Zn–O stretching vibration, which serves as confirmation of the material being zinc oxide. The ZnO-NRNs demonstrated impressive photo-degradation activity, with a 97% degradation rate observed for Rhodamine B (RhB) dye under UV and natural sun irradiation. This outcome underscores the high photo-stability and reusability of ZnO-NRNs as photo-catalysts. The research findings validate the effectiveness of the proposed eco-friendly synthesis method for ZnO-NRNs, which hold significant potential for various applications, including photo-catalysis. The characterized structural and optical properties of these nanorod needles contribute to our understanding of their behavior and performance in different environments.

## Figures and Tables

**Table 1:** First-order rate constant and half-life of RhB photocatalytic degradation.

	UV Light	Visible Light
Rate constants (min^−1^)	0.023844	0.005179
Half-life (units)	29	134

**Table 2:** Evaluating the efficacy of the present photocatalysts in comparison to literature-reported values for ZnO-based photocatalysts.

Methods of Synthesis of ZnONPs	Concentrations of Dye and ZnONPs	Light Sources and Time	Decolorization Percentage (%) of Dye
Sol-gel method	20 mg/L dye and 0.3 g/L of ZnONPs photocatalyst	UV lamp—~150 min	[72] 88% for Congo red, 73% for Acid Blue and 70% for Coomassie Brilliant Blue R-250
Precipitation method	0.025 mg/120 mL distilled water of Orange G Orange G, and 5 mg of ZnONPs	Sunlight light—48 h	[73] 82%
Electrochemical method	100 mg of ZnONPs in 100 mL of dye solution	UV irradiation	[74] 92%
Gel combustion method	10 mg·L^−1^ of methyl orange, and 50 mg of La_x_Zn_1−x_O	Visible light—150 min	[75]~85%
Green synthesis method	Methyl Blue was tested at concentrations of 0.06 mg/mL and 0.03 mg/mL in combination with two different concentrations of ZnO nanoparticles (0.2 mg/mL and 0.1 mg/mL)	UV light radiation-about 40 min	[76] The higher concentration of ZnO nanoparticles (0.2 mg/mL) resulted in a degradation of 92% in the dye, whereas the lower concentration of ZnO (0.1 mg/mL) led to a reduced dye degradation of 83%
*Cymbopogon Proximus grass* extract (this study)	10 mg/L of RhB dye and 0.1 mg of ZnONPs	Natural sunlight for 16 h or ultraviolet light for 160 min	97%

## Data Availability

Data are contained within the article.
